# Bayesian Analysis of Dynamic Cumulative Residual Entropy for Lindley Distribution

**DOI:** 10.3390/e23101256

**Published:** 2021-09-27

**Authors:** Abdullah M. Almarashi, Ali Algarni, Amal S. Hassan, Ahmed N. Zaky, Mohammed Elgarhy

**Affiliations:** 1 Statistics Department, Faculty of Science, King Abdulaziz University, Jeddah 21551, Saudi Arabia; aalmarashi@kau.edu.sa (A.M.A.); ahalgarni@kau.edu.sa (A.A.); 2 Faculty of Graduate Studies for Statistical Research, Cairo University, Giza 12613, Egypt; amal52_soliman@cu.edu.eg; 3 Institute of National Planning, Cairo 11765, Egypt; ahmed.nasser@inp.edu.eg; 4 The Higher Institute of Commercial Sciences, Al Mahalla Al Kubra, Algarbia 31951, Egypt

**Keywords:** Rényi entropy, Lindley distribution, Bayesian estimators, squared error loss function

## Abstract

Dynamic cumulative residual (DCR) entropy is a valuable randomness metric that may be used in survival analysis. The Bayesian estimator of the DCR Rényi entropy (DCRRéE) for the Lindley distribution using the gamma prior is discussed in this article. Using a number of selective loss functions, the Bayesian estimator and the Bayesian credible interval are calculated. In order to compare the theoretical results, a Monte Carlo simulation experiment is proposed. Generally, we note that for a small true value of the DCRRéE, the Bayesian estimates under the linear exponential loss function are favorable compared to the others based on this simulation study. Furthermore, for large true values of the DCRRéE, the Bayesian estimate under the precautionary loss function is more suitable than the others. The Bayesian estimates of the DCRRéE work well when increasing the sample size. Real-world data is evaluated for further clarification, allowing the theoretical results to be validated.

## 1. Introduction

Reference [1] introduced the idea of the Rényi entropy as a measure of randomness for Y. The Rényi entropy can be used to estimate the uncertainty in a random observation. In the study of quantum systems, quantum communication protocols, and quantum correlations [2,3], it has been extensively utilized. The probability density function (PDF) g (.) and the distribution function (CDF) G(.) of the Rényi entropy with the order β is given by

(1) $$\Xi (\beta )={\left(1-\beta \right)}^{-1}\;\;\log\left(\int_{-\infty }^{\infty }g^{\beta }(y)dy\right),\;\;\beta >0,\;\beta \neq 1\;.$$

In recent times, several authors studied the statistical inferences for the entropy measures using different distributions and sampling schemes (for example, [4,5,6,7,8,9,10,11,12]).

Alternative measurements of uncertainty for probability distributions in recent times are of interest to many authors, especially in reliability and survival analysis studies. Therefore, the cumulative residual entropy and its dynamic version have been proposed, respectively, in [13,14]. The DCRRéE is defined as follows:

(2) $$\gamma_{R}(\beta )={\left(1-\beta \right)}^{-1}\log\;\left(\int_{t}^{\infty }\frac{{\bar{G}}^{\beta }(y)}{{\bar{G}}^{\beta }(t)}dy\right),\;\;\beta >0,\;\;\beta \neq 1.$$

where $\bar{G}(t)=1-G(t)$ is the survival function (SF), and for *t* = 0, the DCRRéE leads to the cumulative residual Rényi entropy. In the literature, few works have been regarded for the inferential procedures of DCR entropy for lifetime distributions. Properties of the DCR entropy from the order statistics were presented in [15]. The cumulative residual and past inaccuracy have been proposed in [16] as extensions of the cumulative entropies for the truncated random variables. The Bayesian estimators of the DCR entropy of the Pareto model using different sampling schemes have been studied in [17,18,19]. The Bayesian inference of the DCR entropy for the Pareto II distribution was given in [20]. The Bayesian and non-Bayesian estimators of the DCR entropy for the Lomax distribution were provided in [21].

Reference [22] was the first to use the Lindley distribution to evaluate failure time data, particularly in reliability modeling. It is also a good alternative to the exponential distribution since it combines the exponential and gamma distributions. Hazard rates might be increasing, decreasing, uni-modal, or bathtub-shaped, resulting in the modeling of multiple lifetime data. The PDF of the Lindley distribution is

(3) $$g(y;\theta )=\frac{\theta^{2}}{\theta +1}(1+y)\;e^{-\theta y}\;;\;\;\;y,\;\theta >0.$$

The CDF and the SF of the Lindley distribution are given by

(4) $$G(y;\theta )=1-e^{-\theta y}[1+\frac{\theta y}{\theta +1}];\;\;\;y,\;\theta >0,$$

and

(5) $$\bar{G}(y;\theta )=e^{-\theta y}[1+\frac{\theta y}{\theta +1}];\;\;\;y,\;\theta >0.$$

The authors of [23,24] handled the properties and the inferential procedure for the Lindley distribution. As a result, numerous writers have utilized the Lindley distribution to predict lifetime data under intended circumstances; see [25,26,27,28,29,30] and the references listed therein.

To generate random numbers from the Lindley distribution, we may use the fact that the distribution, as given in Equation (3), is a mixture of exponential (*θ*) and gamma (2, *θ*), with mixing proportions (*θ*/1 + *θ*) and (1/1 + *θ*), respectively. For generating a random sample of size *n*, we have the following simulation algorithm:

(i) Generate $U_{i}$ from uniform (0, 1), *i* = 1, 2, …, *n*.
(ii) Generate $Q_{i}$ from exponential (*θ*), *i* = 1, 2, …, *n*.
(iii) Generate $V_{i}$ from gamma (2, *θ*), *i* = 1, 2, …, *n*.
(iv) If $U_{i}\leq \;\theta /1+\theta$ then set $X_{i}=Q_{i},$ otherwise, set $X_{i}=V_{i}$.

Since the last decade, the Lindley distribution has attracted the attention of researchers for its use in several fields as well as for modeling lifetime data. Herein, we intend to discuss the Bayesian inference of the DCRRéE for the Lindley model. The Bayesian estimators and the Bayesian credible intervals of the DCRRéE under the gamma prior are derived. The proposed estimators are obtained via the squared error (SE), linear exponential (LINEx), and precautionary (PR) loss functions. The Markov Chain Monte Carlo (MCMoC) simulation is utilized because the DCRRéE’s Bayesian estimator is complicated. A real data analysis is given for illustration. We outline the paper as follows: Section 2 gives the formula for the DCRRéE of the Lindley distribution; Section 3 offers the DCRRéE’s Bayesian estimator of the Lindley distribution under the specific loss functions; a description of MCMoC is provided in Section 4; and in Section 5, a real-world data application is shown. Using the findings of our numerical investigations, we came to certain conclusions.

## 2. Expression of the DCRRéE for the Lindley Distribution

This section presents the formula of the DCRRéE for the Lindley distribution. The DCRRéE of the Lindley distribution is obtained by substituting Equation (5) into Equation (2) as follows:

(6) $$\gamma_{R}(\beta )=\frac{1}{\left(1-\beta \right)}\log\;\left(\frac{1}{{\bar{G}}^{\beta }(t)}\int_{t}^{\infty }e^{-\theta \beta y}{\left(1+\frac{\theta y}{\theta +1}\right)}^{\beta }\right)=\frac{1}{\left(1-\beta \right)}\log\;\left(\frac{1}{{\bar{G}}^{\beta }(t)}I\right),$$

where $I=\int_{t}^{\infty }e^{-\theta \beta y}{\left(1+\frac{\theta y}{\theta +1}\right)}^{\beta }dy.$ To obtain *I*, we use the transformation $x=1+\frac{\theta y}{\theta +1}$, then we have

(7) $$I=\left(1+\frac{1}{\theta }\right)\int_{1+\frac{\theta t}{\theta +1}}^{\infty }e^{-\beta \left(1+\theta \right)\left(x-1\right)}x^{\beta }dx=\left(1+\frac{1}{\theta }\right)e^{\beta \left(1+\theta \right)}\int_{1+\frac{\theta t}{\theta +1}}^{\infty }x^{\beta }e^{-\beta \left(1+\theta \right)x}dx.$$

Let z=β(1+θ)x, and then Equation (7) can be expressed as

(8) $$I=\left(1+\frac{1}{\theta }\right)\frac{e^{\beta \left(1+\theta \right)}}{{\left[\beta \left(1+\theta \right)\right]}^{\beta +1}}\int_{\beta \left(1+\theta +\theta t\right)}^{\infty }z^{\beta }e^{-z}dz=\frac{e^{\beta A(\theta )}\Gamma \left(\beta +1,\beta \;A(\theta )\right)}{\theta \beta {\left[A(\theta )\right]}^{\beta }},$$

where Γ(.) stands for an incomplete gamma function and A(θ)=1+θ+θt. By substituting Equation (8) into Equation (6), the DCRRéE for the Lindley distribution is expressed as follows

(9) $$\gamma_{R}(\beta )=\frac{1}{1-\beta }\log\;\left(\frac{e^{\beta A(\theta )}\Gamma \left(\beta +1,\beta \;A(\theta )\right)}{\theta \beta {\left[A(\theta )\right]}^{\beta }}\right).$$

The DCRRéE requires this phrase for the Lindley distribution.

## 3. The Bayesian Estimation

Herein, the Bayesian estimator of the DCRRéE for the Lindley distribution is obtained using the gamma prior. The Bayesian estimator is derived under the selected loss functions, and the Bayesian credible intervals are computed.

A random sample of size *n* taken from the PDF (3) and the CDF (4) can be used if θ is unknown. Then, the likelihood function of the Lindley distribution given the sample $\underline{y}=(y_{1},\ldots ,y_{n}),$ is given by

$$l\left(\theta |\underline{y}\right)=\frac{\theta^{2n}}{{\left(\theta +1\right)}^{n}}e^{-\theta \sum_{i=1}^{n}y_{i}}\prod_{i=1}^{n}\left(1+y_{i}\right).$$

Let us assume that the prior of θ has a gamma distribution with the parameters (*a*, *b*) with the following PDF

$$\pi (\theta )=\frac{a^{b}}{\Gamma (b)}\;\theta^{b-1}e^{-\theta \;a},\;\;\;\;\;\;a,b>0.$$

This is how the posterior PDF of θ given the data can be expressed as

(10) $$\pi (\theta |\underline{y})=k\frac{a^{b}\;\theta^{2n+b-1}}{{\left(\theta +1\right)}^{n}\Gamma (b)}exp\left\{\sum_{i=1}^{n}\ln\left(1+y_{i}\right)-\theta \sum_{i=1}^{n}y_{i}-a\theta \right\},\;\;$$

where

$$k^{-1}=\int_{0}^{\infty }\frac{a^{b}\;\theta^{2n+b-1}}{{\left(\theta +1\right)}^{n}\Gamma (b)}exp\left\{\sum_{i=1}^{n}\ln\left(1+y_{i}\right)-\theta \sum_{i=1}^{n}y_{i}-a\theta \right\}d\theta .\;$$

The Bayes estimator of $\gamma_{R}(\beta )$ under the SE loss function, denoted by ${\hat{\gamma }}_{SE}(\beta )$, is obtained as follows:

(11) $$\begin{array}{l} {\hat{\gamma }}_{SE}(\beta )=\int_{0}^{\infty }\gamma_{R}(\beta )\pi (\theta |\underline{y})\;d\theta \\ \;\;\;\;\;\;\;\;\;\;\;\;\;\;\;=\frac{k}{1-\beta }\int_{0}^{\infty }\log\;\left(\frac{e^{\beta A(\theta )}\Gamma \left(\beta +1,\beta \;A(\theta )\right)}{\theta \beta {\left[A(\theta )\right]}^{\beta }}\right)\frac{a^{b}\;\theta^{2n+b-1}}{{\left(\theta +1\right)}^{n}\Gamma (b)}exp\left\{\sum_{i=1}^{n}\ln\left(1+y_{i}\right)-\theta \sum_{i=1}^{n}y_{i}-a\theta \right\}d\theta . \end{array}$$

Based on the LINEx loss function, the Bayes estimator of $\gamma_{R}(\beta )$ says ${\hat{\gamma }}_{LINEx}(\beta )$ is given by

(12) $$\begin{array}{l} {\hat{\gamma }}_{LINEx}(\beta )=\frac{-1}{\nu }\ln[\int_{0}^{\infty }e^{-v\;\gamma_{R}(\beta )}\;\;\pi (\theta |\underline{y})\;d\theta ],\;\;\nu \neq 0,\\ \;\;\;\;\;\;\;\;\;\;\;\;\;\;\;=\frac{-1}{\nu }\ln\left[k\int_{0}^{\infty }{\left(\frac{e^{\beta A(\theta )}\Gamma \left(\beta +1,\beta \;A(\theta )\right)}{\theta \beta {\left[A(\theta )\right]}^{\beta }}\right)}^{\frac{-v}{\beta -1}}\frac{a^{b}\;\theta^{2n+b-1}}{{\left(\theta +1\right)}^{n}\Gamma (b)}exp\left\{\sum_{i=1}^{n}\ln\left(1+y_{i}\right)-\theta \sum_{i=1}^{n}y_{i}-a\theta \right\}d\theta \right]. \end{array}$$

Using the PR loss function, the Bayes estimator of $\gamma_{R}(\beta )$ says ${\hat{\gamma }}_{PR}(\beta )$ is given by

(13) $$\begin{array}{l} {\hat{\gamma }}_{PR}(\beta )={\left[\int_{0}^{\infty }{\left(\gamma_{R}(\beta )\right)}^{2}\;\;\pi (\theta |\underline{y})\;d\theta \right]}^{0.5}\\ \;\;\;\;\;\;\;\;\;\;\;\;\;={\left[\frac{k}{1-\beta }\int_{0}^{\infty }{\left\{\log\;\left(\frac{e^{\beta A(\theta )}\Gamma \left(\beta +1,\beta \;A(\theta )\right)}{\theta \beta {\left[A(\theta )\right]}^{\beta }}\right)\right\}}^{2}\frac{a^{b}\;\theta^{2n+b-1}}{{\left(\theta +1\right)}^{n}\Gamma (b)}exp\left\{\sum_{i=1}^{n}\ln\left(1+y_{i}\right)-\theta \sum_{i=1}^{n}y_{i}-a\theta \right\}d\theta \right]}^{0.5}. \end{array}$$

As previously stated, the analytical solution to Integrations (11–13) is extremely difficult to acquire due to complex mathematical forms. To approximate these integrations, the MCMoC technique is used. Furthermore, using the method described in [31], we obtain the Bayesian credible intervals of $\gamma_{R}(\beta ).$ A credible interval is the Bayesian equivalent of a confidence interval. The upper (U) and lower (L) credible limits are the U and L endpoints of a credible interval, respectively.

The probability that a credible interval will contain the unknown parameter θ is called the “confidence coefficient”. If we suppose the L and U credible limits, respectively, for the parameter θ, then P (L<θ<U)=1−η, where (1−η) 10000 is the confidence coefficient.

## 4. Numerical Illustrations and Results

For the Lindley distribution at β=0.5, a numerical analysis is conducted in this part to examine the performance of the Bayesian estimates of $\gamma_{R}(\beta ).$ In Bayesian literature, the Metropolis–Hastings (MH) algorithm (see [32]) is one of the most well-known subclasses of the MCMoC technique for simulating deviations from the posterior density and producing good approximation results. MCMoC simulations are run for selected sample sizes and loss functions. R 4.1.1 will be used to run the MH algorithm.

The MCMoC method is used to generate samples from the posterior distributions and then to compute the DCRRéE’s Bayesian estimators under the intended loss functions. MCMoC schemes come in a wide range of options. Gibbs sampling and the more general Metropolis-within-Gibbs samplers are a significant subclass of the MCMoC methods.

To pull samples from the posterior density functions and then compute the Bayesian estimators, we use the following MCMoC technique, see Algorithm 1.

**Algorithm 1:** Algorithm of MCMC**Step 1.** Set initial value of *θ* as $\theta^{\left(0\right)}$.**Step 2.** For *i* = 1, 2, …, N = 1000 repeat the following steps:   2.1: Set $\theta =\theta {\;}^{\left(i-1\right)}.$   2.2: Generate a new candidate parameter value $\overset{`}{\theta }\;\mathrm{from}\;N\;(\theta ,S_{\theta }).$
   2.3: Generate $r=min\left(\frac{\pi (\overset{`}{\theta }\;|x)}{\pi (\theta \;|x)},\;1\right)$, where π(·) is the posterior density in Equation (10).   2.4: Generate a sample *u* from the uniform distribution U (0, 1).
   2.5: Accept or reject the new candidate $\overset{`}{\theta }.$
$$\{\begin{array}{c} If\;u\;\leq \;r\;set\theta {\;}^{\left(i\right)}=\overset{`}{\theta }\;\\ otherwise\;set\theta {\;}^{\left(i\right)}=\theta . \end{array}$$
**Step 3.** Obtain the Bayesian estimator of *θ* and compute the DCRRéE function $\gamma_{R}(\beta )$ with respect to the loss functions as follows:
$${\hat{\gamma }}_{R}(\beta )=\frac{1}{N-M}\sum_{i=M+1}^{N}\gamma_{R}(\beta ,\theta^{\left(i\right)})$$
where M = 0.2 N is the burn-in period. We also found that the acceptance rate is equal to 0.85.The formulas of relative absolute biases (RABs) and the estimated risks (ERs) are given
$$\mathrm{RABs}=\frac{\sum_{i=M+1}^{N}\left|{\hat{\gamma }}_{i}(\beta )-\gamma_{i}(\beta )\right|}{M-N}\;\;\;\mathrm{and}\mathrm{ERs}=\frac{\sum_{i=M+1}^{N}{\left({\hat{\gamma }}_{i}(\beta )-\gamma_{i}(\beta )\right)}^{2}}{M-N}.$$

The hyper-parameters of the gamma distribution are specified as *a* = 2 and *b* = 1. Choose v = (−1, 1) for the LINEx loss function, which represents underestimation and overestimation, respectively. Using a sample size of 5,000, *n* = 30, 50, 70, and 100 are generated from the Lindley model. The true values of the parameter values are chosen as θ= (0.8, 1.5 , 2). The actual value of the DCRRéE measure is elected as $\gamma_{R}(\beta )=\;2.433289127,\;1.025114899.\;0.38237199\;$ where *t* = 0.5, and $\gamma_{R}(\beta )=\;2.31065,\;0.90832.\;\;0.27434$ where *t* = 1.5. Measures including the RABs and the ERs of the Bayes estimates (Bes) of the DCRRéE, along with the width (WD) of the Bayesian credible interval, are computed.

### 4.1. Numerical Results

**Figure 1:**
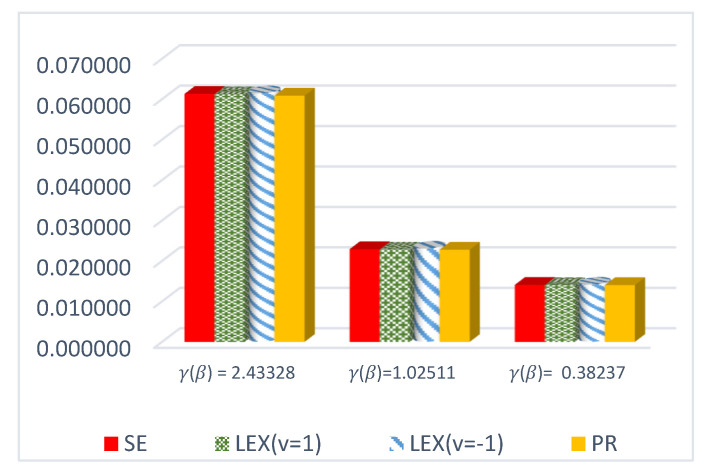
ER of DCRRéE estimates under proposed loss functions at *n* = 30 and *t* = 0.5.

**Figure 2:**
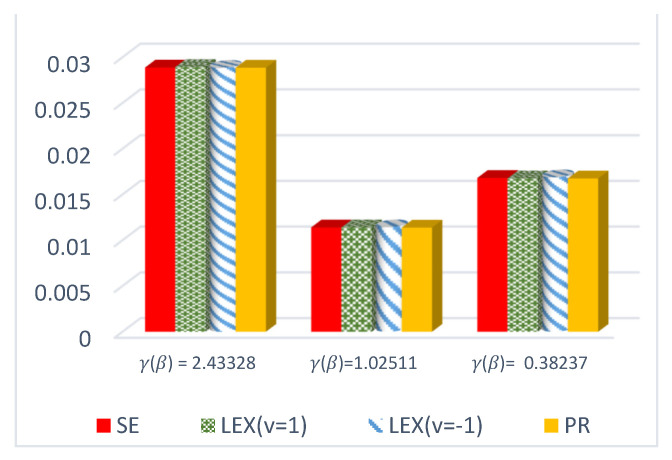
ER of DCRRéE estimates under proposed loss functions at *n* = 100 and *t* = 0.5.

**Figure 3:**
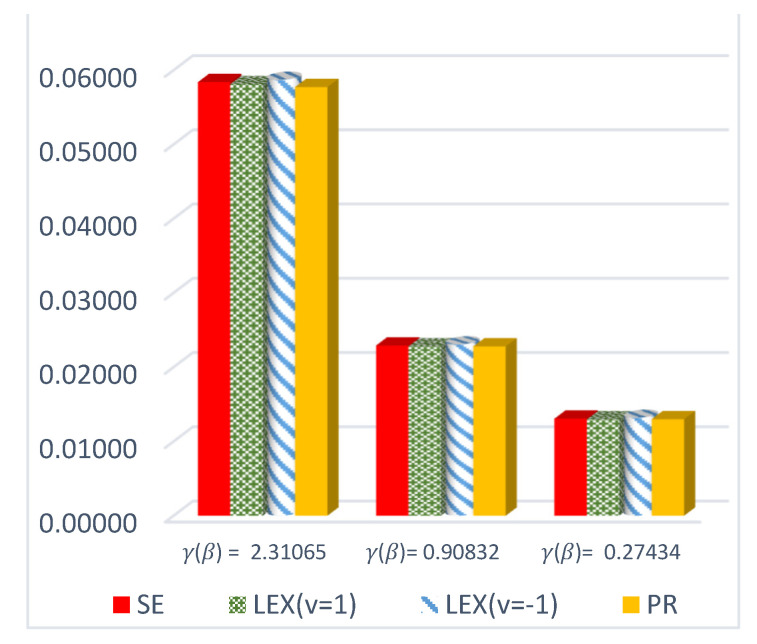
ER of DCRRéE estimates under proposed loss functions at *n* = 30 and *t* = 1.5.

**Figure 4:**
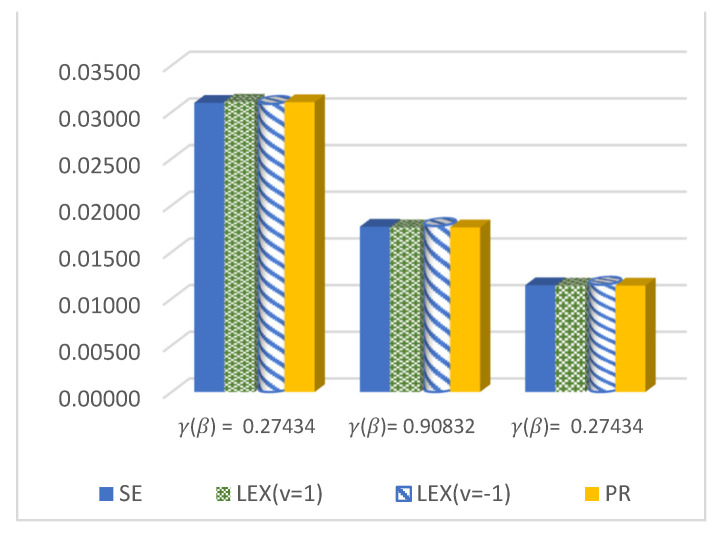
ER of DCRRéE estimates under proposed loss functions at *n* = 100 and *t* = 1.5.

The results of this study are presented in Table 1, Table 2 and Table 3 for the DCRRéE estimates at *t* = 0.5, and Table 4, Table 5 and Table 6 give the simulation results for the DCRRéE estimates at *t* = 1.5. Figure 1, Figure 2, Figure 3 and Figure 4 also provide the numerical results. Accordingly, we may draw the following conclusions about the DCRRéE estimates.

- As the θ value grows, the DCRRéE estimates appear smaller for a similar value of *t*.
- The DCRRéE estimates decrease with an increasing value of *t* for a similar value of θ.
  At *t* = 0.5, the following notes can be recorded:
- The estimated risk of ${\hat{\gamma }}_{LINEx}(\beta )$ at v = −1 picks the lowest values for *n* = 50 and 70 while the estimated risk of ${\hat{\gamma }}_{LINEx}(\beta )$ at v = 1 picks the lowest values at *n* = 100. In addition, the width of the credible interval for ${\hat{\gamma }}_{LINEx}(\beta )$ at v = −1 takes the lowest values for *n* = 100 (see Table 1).
- The estimated risk of ${\hat{\gamma }}_{PR}(\beta )$ has the lowest values for all *n* values, and the width of the credible interval for ${\hat{\gamma }}_{PR}(\beta )$ picks the lowest values for all values of *n* except *n* = 70 (see Table 2).
- At actual value $\gamma_{R}(\beta )=\;0.38237199\;(\theta =2.0),$ the estimated risk of ${\hat{\gamma }}_{LINEx}(\beta )$ at v = 1 for all *n* values except at *n* = 100 has the lowest values. Moreover, the width of the credible interval for ${\hat{\gamma }}_{LINEx}(\beta )$ at v = 1 obtains the lowest value at *n* = 70 (see Table 3).
- We can see from Figure 1 that the estimated risk for ${\hat{\gamma }}_{PR}(\beta )$ at the true value $\gamma_{R}(\beta )>1$ for *n =* 30 has the lowest values when compared to the other estimates, except at the true value of $\gamma_{R}(\beta )=\;0.38237.$
- Figure 2 indicates that the estimated risks of ${\hat{\gamma }}_{LINEx}(\beta )$ at v = 1 have the lowest value at $\gamma_{R}(\beta )=\;2.43328$ when compared to the other estimates for *n* = 100.

**Figure 5:**
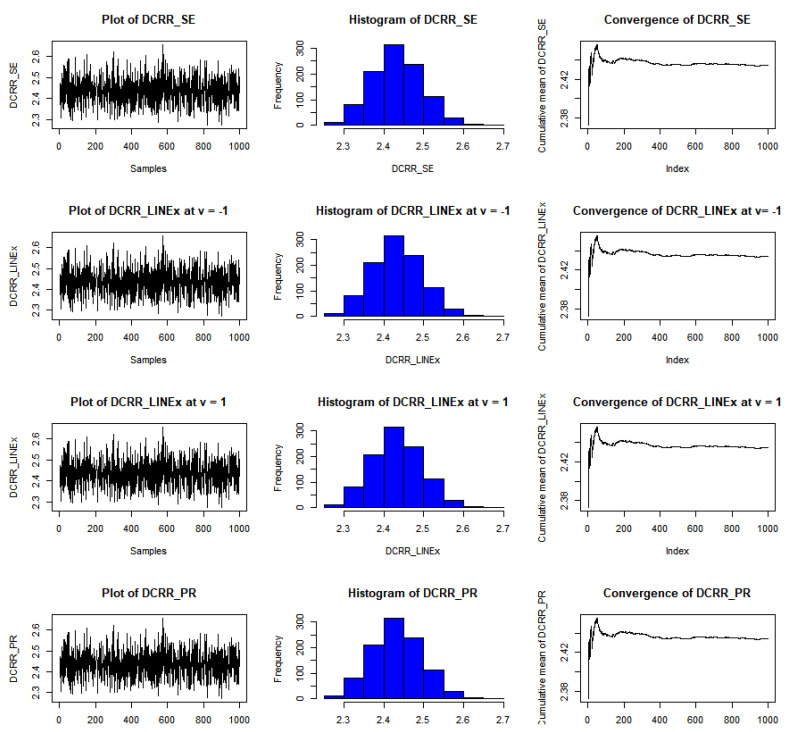
Example of convergence of MCMoC of estimates for $\gamma_{R}(\beta )$, at *t* = 0.5, *θ* = 0.8, and *n* = 30.

**Figure 6:**
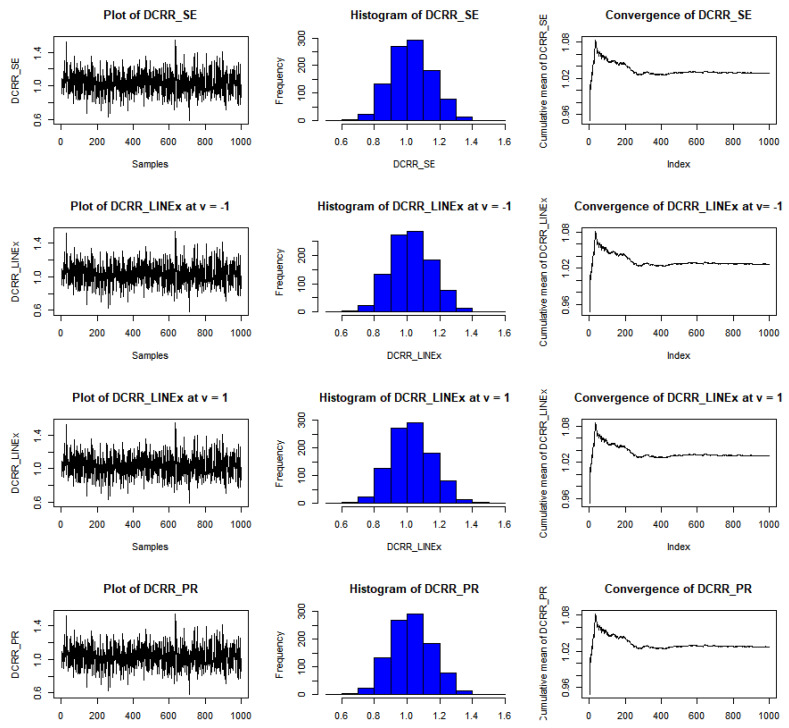
Example of convergence of MCMoC estimates for $\gamma_{R}(\beta )$, at *t* = 0.5, *θ* = 1.5, and *n* = 100.

**Figure 7:**
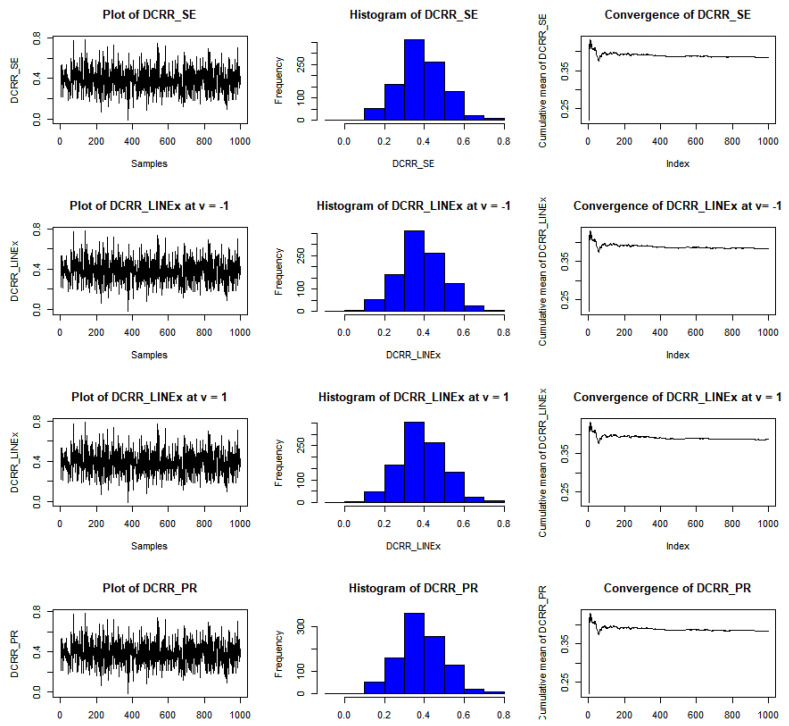
Example of convergence of MCMoC estimates for $\gamma_{R}(\beta )$, at *t* = 0.5, *θ* = 2.0, and *n* = 50.

The following are the notes that may be found at *t* = 1.5:

- The estimated risk of ${\hat{\gamma }}_{LINEx}(\beta )$ at v = −1 obtains the lowest values at *n* = 70 and 100 while the estimated risk of ${\hat{\gamma }}_{PR}(\beta )$ has the lowest values for *n* = 30 and 50. The width of the Bayesian credible interval for ${\hat{\gamma }}_{LINEx}(\beta )$ at v = −1 is the smallest in comparison with other estimates for *n* = 50 and 70 (see Table 4).
- At *n* = 30 and 100, the estimated risk of ${\hat{\gamma }}_{PR}(\beta )$ has the lowest values, while the estimated risk of ${\hat{\gamma }}_{SE}(\beta )$ has the lowest values at *n* = 50 and 70. The width of the Bayesian credible interval for ${\hat{\gamma }}_{LINEx}(\beta )$ at v = 1 is the shortest compared to the others via the SE and PR loss functions, except at *n* = 100 (see Table 5).
- We can see from Figure 3 that the estimated risk of ${\hat{\gamma }}_{PR}(\beta ),$ at *n =* 30 holds the lowest values for all actual values of $\gamma_{R}(\beta ),$, except at $\gamma_{R}(\beta )=\;0.27434.$
- For a large sample size (*n* = 100), the estimated risks for ${\hat{\gamma }}_{LINEx}(\beta )$ at v = 1 obtain the lowest value at actual value of $\gamma_{R}(\beta )=\;0.27434,\;\;0.90832$, as shown in Figure 4.
- We conclude from Table 6 that the estimated risks of ${\hat{\gamma }}_{LINEx}(\beta )$ at v = 1 provide the lowest values for all values of *n.* Moreover, the width of the Bayesian credible intervals for ${\hat{\gamma }}_{LINEx}(\beta )$ at v = 1 takes the lowest values with respect to all possible values of *n,* except at *n* = 30 and 70.
- Figure 5, Figure 6 and Figure 7 represent trace plots, histograms, and convergences for $\gamma_{R}(\beta )$ estimates using the MH algorithm.

### 4.2. Application

**Figure 8:**
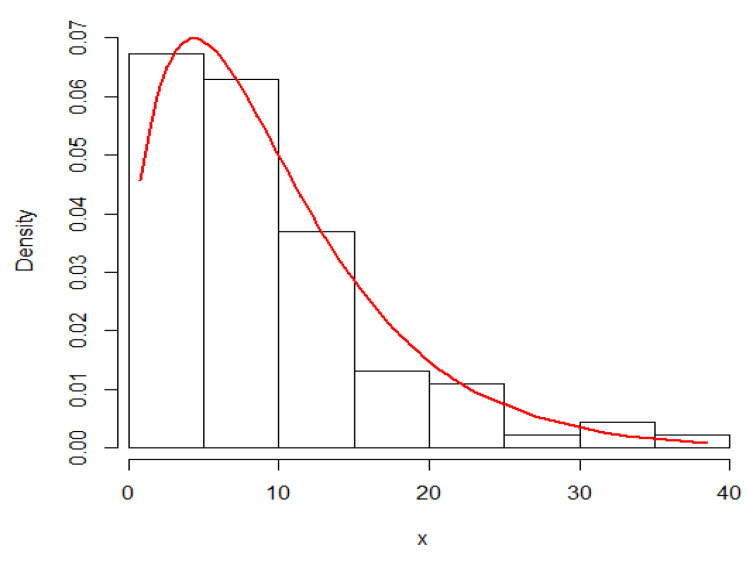
Fitted PDF plots of Lindley model for the data set.

**Figure 9:**
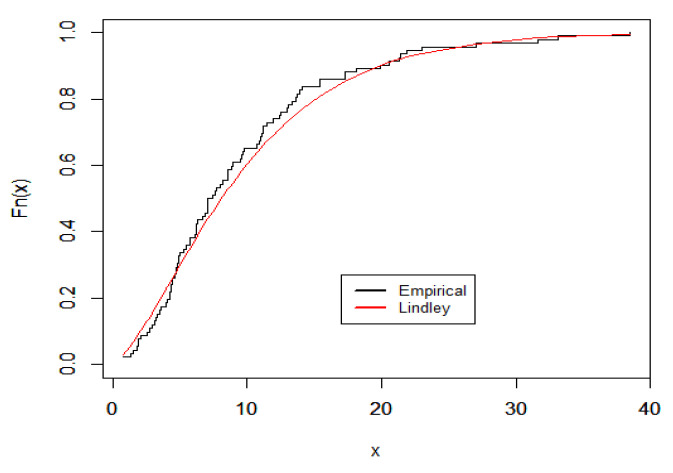
Fitted CDF plots of Lindley model for the data set.

Here, we demonstrate the technique described in the preceding section by using an actual data set that represents the waiting times (in minutes) before receiving service for 100 bank customers. Reference [23] discussed the detailed statistics that showed the data fitted the Lindley distribution. Figure 8 and Figure 9 provide plots of fitted PDF and CDF for the data under consideration. The Bayes estimates of the DCRRéE at *t* = 0.5 and 1.5 at the intended loss functions are reported in Table 7.

As expected, the DCRRéE estimators for the proposed loss functions decrease with time, as seen in this example.

## 5. Concluding Remarks

The Bayesian estimators of the DCRRéE for the Lindley distribution are investigated in this study. The Bayesian estimators of the DCRRéE for the Lindley model are thought to be produced by both symmetric and asymmetric loss functions. The MCMoC method is used to calculate the Bayesian estimator and the Bayesian credible intervals. The behavior of the DCRRéE estimators for the Lindley distribution is evaluated using some precision criteria. Real-world data and simulation concerns are addressed. Regarding the outcomes of the study, we conclude that for small actual values of the DCRRéE, the estimated risk and width of the Bayesian credible intervals of the DCRRéE estimates under the linear exponential loss function are often fewer than those based on the squared error and precautionary loss functions. At *t* = 0.5, the width of the Bayesian credible intervals for the DCRRéE estimates via the linear exponential loss function is less than the others via the squared error and precautionary loss functions for a sample size of large values and large actual values of the DCRRéE. However, at *t* = 1.5, the width of the Bayesian credible interval for the DCRRéE estimates via the precautionary loss function is smaller than the equivalent estimates via the squared error and linear exponential loss functions. For small DCRRéE values, the Bayesian estimates via the linear exponential loss function are preferable to other estimates under the squared error and precautionary loss functions. However, for a high true value of the DCRRéE, the Bayesian estimates under the precautionary loss function are preferable to the other estimates via the loss functions chosen.

## Figures and Tables

**Table 1:** Measures of Accuracy for DCRRéE at θ = 0.8, t = 0.5, and $\gamma_{R}(\beta )=\;2.433289$.

*n*	SE				LINEx (v = 1)				LINEx (v = −1)				PR			
	BE	RAB	ER	WD	BE	RAB	ER	WD	BE	RAB	ER	WD	BE	RAB	ER	WD
30	2.42926	0.00166	0.06132	0.94796	2.42550	0.00320	0.06121	0.94596	2.43302	0.00011	0.06146	0.95129	2.42450	0.00361	0.06088	0.94389
50	2.42149	0.00485	0.04538	0.78382	2.41824	0.00618	0.04552	0.78713	2.42475	0.00351	0.04528	0.78514	2.41744	0.00651	0.04537	0.78679
70	2.41950	0.00567	0.03941	0.75725	2.41658	0.00687	0.03951	0.75845	2.42242	0.00447	0.03934	0.75966	2.41587	0.00716	0.03939	0.75794
100	2.43023	0.00126	0.02889	0.66839	2.42763	0.00233	0.02894	0.66955	2.43284	0.00018	0.02885	0.66541	2.42699	0.00259	0.02887	0.66919

**Table 2:** Measures of Accuracy for DCRRéE at θ=1.5, t=0.5, and $\gamma_{R}(\beta )=1.02511$.

*n*	SE				LINEx (v = 1)				LINEx (v = −1)				PR			
	BE	RAB	ER	WD	BE	RAB	ER	WD	BE	RAB	ER	WD	BE	RAB	ER	WD
30	1.02767	0.00250	0.02292	0.59123	1.02535	0.00023	0.022793	0.59016	1.03000	0.00477	0.02306	0.59175	1.02610	0.00096	0.022787	0.59015
50	1.03075	0.00550	0.02128	0.56636	1.02856	0.00336	0.02123	0.56555	1.03295	0.00764	0.02135	0.56662	1.02928	0.00406	0.02121	0.56545
70	1.01934	0.00563	0.02024	0.54244	1.01716	0.00776	0.02025	0.53970	1.02153	0.00350	0.02025	0.54470	1.01788	0.00706	0.02021	0.54031
100	1.02838	0.00319	0.01682	0.48992	1.02636	0.00121	0.01676	0.49011	1.03041	0.00516	0.01689	0.49149	1.02702	0.00186	0.01675	0.48942

**Table 3:** Measures of Accuracy for DCRRéE for θ=2.0, t=0.5, and $\gamma_{R}(\beta )=\;0.38237$.

*n*	SE				LINEx (v = 1)				LINEx (v = − 1)				PR			
	BE	RAB	ER	WD	BE	RAB	ER	WD	BE	RAB	ER	WD	BE	RAB	ER	WD
30	0.39342	0.02888	0.01409	0.45858	0.39158	0.02408	0.01403	0.45810	0.39525	0.03368	0.01415	0.45534	0.39249	0.02646	0.01404	0.45710
50	0.38557	0.00835	0.01334	0.43146	0.38381	0.00377	0.01330	0.43208	0.38732	0.01294	0.01339	0.43187	0.38468	0.00604	0.01331	0.43033
70	0.38513	0.00721	0.01254	0.42933	0.38347	0.00287	0.01252	0.42705	0.38679	0.01156	0.01257	0.42750	0.38429	0.00503	0.01257	0.42820
100	0.38601	0.00952	0.01141	0.41235	0.38437	0.00522	0.01140	0.41399	0.38766	0.01383	0.01142	0.41399	0.38519	0.00736	0.01139	0.41346

**Table 4:** Measures of Accuracy for DCRRéE for θ=0.8, t=1.5, and $\gamma_{R}(\beta )=\;2.31065$.

*n*	SE				LINEx (v = 1)				LINEx (v = −1)				PR			
	BE	RAB	ER	WD	BE	RAB	ER	WD	BE	RAB	ER	WD	BE	RAB	ER	WD
30	2.34019	0.01279	0.05839	0.92243	2.33632	0.01111	0.05811	0.92058	2.34407	0.01446	0.05871	0.91961	2.33524	0.01064	0.05774	0.91789
50	2.29427	0.00709	0.04883	0.86754	2.29102	0.00850	0.04888	0.86787	2.29753	0.00568	0.04880	0.86563	2.29020	0.00885	0.04869	0.86675
70	2.29300	0.00764	0.03709	0.74947	2.29013	0.00888	0.03725	0.75022	2.29586	0.00640	0.03695	0.74925	2.28945	0.00918	0.03717	0.74970
100	2.28540	0.01093	0.03098	0.65564	2.28284	0.01204	0.03112	0.65423	2.28797	0.00982	0.03087	0.65897	2.28222	0.01230	0.03106	0.65275

**Table 5:** Measures of Accuracy for DCRRéE for θ=1.5, t=1.5, and $\gamma_{R}(\beta )=\;0.90832$.

*n*	SE				LINEx (v = 1)				LINEx (v = −1)				PR			
	BE	RAB	ER	WD	BE	RAB	ER	WD	BE	RAB	ER	WD	BE	RAB	ER	WD
30	0.91976	0.01260	0.02292	0.56958	0.91744	0.01005	0.022792	0.56538	0.92208	0.01515	0.02306	0.57188	0.91819	0.01087	0.022788	0.56644
50	0.91339	0.00559	0.02011	0.54160	0.91124	0.00322	0.02092	0.53828	0.91555	0.00796	0.02021	0.54329	0.91194	0.00399	0.02091	0.55795
70	0.89959	0.00961	0.02005	0.54054	0.89749	0.01192	0.02081	0.53003	0.90169	0.00730	0.02011	0.53211	0.89820	0.01114	0.02076	0.54979
100	0.91480	0.00714	0.01771	0.51330	0.91285	0.00499	0.01765	0.51441	0.91675	0.00928	0.01779	0.51429	0.91349	0.00569	0.01764	0.51315

**Table 6:** Measures of Accuracy for DCRRéE at θ=2.0, t=1.5, and $\gamma_{R}(\beta )=\;0.27434$.

*n*	SE				LINEx (v = 1)				LINEx (v = −1)				PR			
	BE	RAB	ER	WD	BE	RAB	ER	WD	BE	RAB	ER	WD	BE	RAB	ER	WD
30	0.28367	0.03401	0.01306	0.43538	0.28192	0.02763	0.01296	0.43471	0.28542	0.04039	0.01318	0.43649	0.28278	0.03077	0.01300	0.43461
50	0.27769	0.01221	0.01285	0.43401	0.27596	0.00591	0.012817	0.43286	0.27942	0.01853	0.01290	0.43440	0.27682	0.00903	0.01282	0.43410
70	0.28249	0.02972	0.01277	0.43031	0.28072	0.02326	0.01272	0.43063	0.28427	0.03620	0.01283	0.42760	0.28160	0.02646	0.01273	0.43075
100	0.28390	0.03486	0.01145	0.41154	0.28225	0.02883	0.01140	0.40894	0.28556	0.04089	0.01150	0.41205	0.28307	0.03182	0.01141	0.41037

**Table 7:** DCRRéE Bayesian estimates at *t* = 0.5 and 1.5 for elected loss functions.

*t*	${\hat{\gamma }}_{SE}(\beta )$	${\hat{\gamma }}_{LINEx}(\beta ),(v=1)$	${\hat{\gamma }}_{LINEx}(\beta ),(v=-1)$	${\hat{\gamma }}_{PR}(\beta )$
0.5	3.711552	3.701186	3.703288	3.708402
1.5	3.638074	3.634769	3.638159	3.635013

## Data Availability

If you would like to obtain the numerical dataset used to conduct the study reported in the publication, please contact the appropriate author.

## Acronyms & Abbreviations

BEs	Bayes estimates
CDF	Cumulative distribution function
DCR	dynamic cumulative residual
DCRRéE	dynamic cumulative residual Rényi entropy
ER	Estimated Risk
LINEx	Linear exponential loss function
L	Lower credible limit
MCMoC	Markov Chain Monte Carlo
MH	Metropolis–Hastings
PDF	Probability density function
PR	Precautionary loss function
RABs	Relative absolute biases
SE	Squared error loss function
SF	Survival function
U	Upper credible limit
WD	Width of credible intervals
